# Exploring functionally annotated transcriptional consensus regulatory elements with CONREL

**DOI:** 10.1093/database/baaa071

**Published:** 2020-11-09

**Authors:** Davide Dalfovo, Samuel Valentini, Alessandro Romanel

**Affiliations:** Laboratory of Bioinformatics and Computational Genomics, Department of Cellular, Computational and Integrative Biology (CIBIO), University of Trento, Via Sommarive 9, 38123 Trento, Italy; Laboratory of Bioinformatics and Computational Genomics, Department of Cellular, Computational and Integrative Biology (CIBIO), University of Trento, Via Sommarive 9, 38123 Trento, Italy; Laboratory of Bioinformatics and Computational Genomics, Department of Cellular, Computational and Integrative Biology (CIBIO), University of Trento, Via Sommarive 9, 38123 Trento, Italy

## Abstract

Understanding the interaction between human genome regulatory elements and transcription factors is fundamental to elucidate the structure of gene regulatory networks. Here we present CONREL, a web application that allows for the exploration of functionally annotated transcriptional ‘consensus’ regulatory elements at different levels of abstraction. CONREL provides an extensive collection of consensus promoters, enhancers and active enhancers for 198 cell-lines across 38 tissue types, which are also combined to provide global consensuses. In addition, 1000 Genomes Project genotype data and the ‘total binding affinity’ of thousands of transcription factor binding motifs at genomic regulatory elements is fully combined and exploited to characterize and annotate functional properties of our collection. Comparison with other available resources highlights the strengths and advantages of CONREL. CONREL can be used to explore genomic loci, specific genes or genomic regions of interest across different cell lines and tissue types. The resource is freely available at https://bcglab.cibio.unitn.it/conrel.

## Introduction

Cis-regulatory elements are genomic regions of DNA that concur to the regulation of the transcription of nearby genes. Promoters initiate transcription of a gene, are located near the transcription start site (TSS) of a gene and encompass relatively short sequences. Enhancers, instead, influence the transcription of genes and can be located upstream, downstream, within the introns or relatively far away from the gene(s) itself.

Transcriptional regulation is usually mediated by interactions of multiple transcription factors (TFs) and knowledge of specific interaction between TFs and regulatory elements is fundamental to understand the topology of gene regulatory networks. Recently, genome-wide chromatin annotations have permitted the mapping of putative regulatory elements across multiple human cell types by combining patterns of different combinations of histone modifications. It has been shown that specific patterns of histone modifications define specific regulatory elements (1–4). Trimethylation of H3 lysine 4 (H3K4me3) occurs at regulatory elements primarily associated with promoters/transcription starts. Instead, monomethylation of H3 lysine 4 (H3K4me1) occurs at regulatory elements associated with enhancers and other distal elements. Acetylation of H3 lysine 27 (H3K27ac) marks active regulatory elements and can be used to distinguish active enhancers from their inactive counterparts.

The Encyclopedia of DNA Elements (ENCODE) (5) and the NIH Roadmap Epigenomics Program (Roadmap) (6) were established with the aim of building and delineating all human genome functional elements. Both projects have performed a large number of sequence-based studies to map functional elements, and all the data they have generated so far, which is mostly derived from ChIP-seq experiments, are publicly accessible through the ENCODE website (www.encodeproject.org), fully incorporated and standardized. Many tools and methods have been developed over the years to explore these ChIP-seq data collections. Most of the available resources allow to explore the landscape of regulatory elements (usually promoters and/or enhancers) by means of single cell-line/tissue histone markers ChIP-seq experiments (7–9) or by their global aggregation and integration (5, 10). In addition, other resources exploit instead TF ChIP-seq data to explore potential TF:DNA interactions (11, 12).

TFs are a class of proteins essential for controlling different genetic programs. TFs directly interpret the genome, bind the DNA at either enhancer or promoter regions in a sequence-specific manner and regulate transcription. TF binding sites are short and usually degenerated sequences. The human genome encodes for thousands of different TFs, which can have more than 1000-fold preference for specific binding sequences compared to other sequences. A single TF can regulate different genes in different cell types indicating that gene regulatory networks are dynamic even within the same organism. DNA-binding specificity for a TF is commonly summarized as a motif model representing the set of genomic sequences bound by the TF. This model is commonly represented as a positional frequency matrix (PFM), which specifies the frequency distribution of the four nucleotides in each position of specific TF binding sites, and is typically used to assign a score that expresses the degree of similarity between the DNA sequence and the TF. While most computational approaches to date predict a TF:DNA interaction when the score derived from the PFM is above a given cutoff, other cutoff-free methods that use PFMs to predict TF binding have been proposed (13, 14). Among these, an effective measure considers the total binding affinity (TBA) of a sequence (15, 16), whereby the affinity of a regulatory element for a specific TF is evaluated considering the whole sequence, hence correctly keeping into account both high- and low-affinity sites.

In this work, we present CONREL, a web application that enables the exploration of regulatory elements across the human genome. Regulatory elements are built using a ‘consensus’ approach by computing the agreement of histone mark annotations across multiple experiments. For each ‘consensus regulatory element’ (CRE), CONREL provides annotations of which TFs have enriched TBAs. While CREs allows to better characterize regulatory elements that are conserved among multiple experiments, cell lines and tissue types, the TBA approach allows to measure a one-to-one relationship between a TF and a CRE.

Briefly, ENCODE peak regions data across sample replicates and multiple experiments for the same cell line are combined, and ‘consensus regions’ for each cell line are computed integrating also TSS data. Then, data of cell lines originated from the same tissue are combined to obtain a landscape of ‘tissue consensus regions’ and data of all cell lines are combined to obtain an overall landscape of ‘global consensus regions’. Finally, tissue and global regions are characterized by identifying all TFs showing enriched TBA and by determining the fraction of common alleles among 1000 Genomes Project individuals that support TFs TBA enrichment. To the best of our knowledge, none of the available resources provide regulatory elements description at multiple levels of abstraction. In addition, a comprehensive landscape of TF TBAs across the human genome regulatory elements is still missing.

CONREL can be hence used to characterize and explore regulatory elements and their functional properties across any genomic locus, gene or genomic region of interest across different cell lines and tissue types.

## Implementation

### Identification of CREs

**Figure 1.**
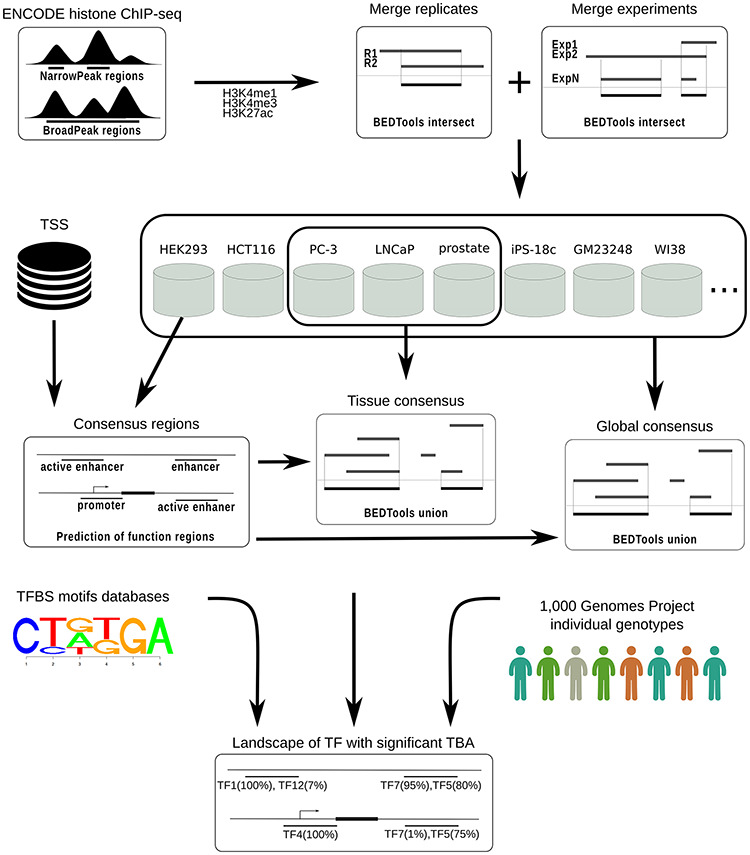
CONREL workflow to generate consensus regions and TBAs annotations.

To identify consensus regions for transcriptional regulatory elements of the human genome, the computational workflow depicted in Figure 1 was implemented and used.

ChIP-seq data from ENCODE (based on hg19 assembly) was downloaded for all cell lines with H3K4me1, H3K4me3 or H3K27ac histone markers peak data available. Data available as of September 2018 was downloaded for both narrowPeak and broadPeak formats.

Peaks from the broadPeak collection were filtered considering only peaks with reported *P*-value smaller than 0.01. No filters were applied to peaks from narrowPeak collection, since all peaks had a *P*-value smaller than 0.01. Peak files were then converted into BrowserExtensibleData (BED) format files in which each peak region is represented with information about the chromosome and the start/end positions of the corresponding genomic region (BED3 format files).

For each marker, peak regions from sample replicates, if available, were merged considering only overlapping regions, retaining hence replicates intersection. Resulting peak regions for different experiments of the same cell line were then combined considering only overlapping regions present in at least two (when available) experiments and retaining the merge of these overlaps. Consensus regions for each cell line were finally computed based on the markers available for that particular cell line, considering three types of regulatory elements. Specifically, consensus regions for ‘promoters’ were defined considering all the regions occupied by H3K4me3, within a window of 1 kb around a TSS. Consensus regions for ‘enhancers’ were defined considering regions occupied by H3K4me1, depleted of H3K4me3, and with distance greater than 1 kb from TSS. Enhancer regions were considered ‘active’ if overlapped by at least one H3K27ac peak region. Note that if, for example, only the marker H3K4me3 was available for a cell line, then only the consensus regions for promoters were computed. All analysis was performed using BEDTools (17) intersect and merge commands with default parameters.

TSS data were downloaded from UCSC Genome Browser (SwitchGear Genomics Transcription Start Sites track) and only TSS with a score ≥ 10 were retained; TSS data with low stringency score (score < 10 by UCSC Genome Browser definition) were excluded.

For all three types of transcriptional regulatory elements considered here, we also characterized tissue and global CREs. Tissue consensuses were computed by merging consensus regions across cell lines that originated from the same tissue, while global consensuses were computed by merging consensus regions across all considered cell lines. In both cases, the consensus was computed considering all regions overlapping in at least two cell lines and retaining the union of the resulting overlapping regions.

CREs were computed considering narrow and broad peak collections separately. A summary of cell lines and tissues for both narrow and broad peak data that were considered in this work is reported in Supplementary Table 1.

### TBA scores at CREs

The TBA is a method used to describe the affinity of a DNA sequence for a TF described by a PFM with a single score, taking into account binding sites of all possible affinities, and weighting them based on a physical model of TF:DNA interactions. TBA was first introduced and applied to study transcriptional regulation in yeast (15, 16) and recently used to study the evolution of cis-regulatory elements in humans (18) and to detect and characterize Expression quantitative trait loci (eQTL) (19, 20).

Formally, the TBA ${a_{rw}}$ of a sequence $r$ for a PFM $w$ is given by:

$$\begin{equation*}{a_{rw}} = \mathop \sum \limits_{i = 1}^{L - l + 1} max\left( {\mathop \prod \limits_{j = 1}^l {{P\left( {{w_j},{r_{i + j - 1}}} \right)} \over {P\left( {b,{r_{i + j - 1}}} \right)}},\mathop \prod \limits_{j = 1}^l {{P\left( {{w_{l - j + 1}},r\,{\prime_{i + j - 1}}} \right)} \over {P\left( {b,r\,{\prime_{i + j - 1}}} \right)}}} \right)\end{equation*}$$

where *l* is the length of the PFM $w,L$ is the length of the sequence $r,{r_i}$ is the nucleotide at the position $i$ of the sequence $r$ on the plus strand, $r\,{\prime_i}$ is the nucleotide in the same position but on the other strand, $P\left( {{w_j},{r_i}} \right)$ is the probability to observe the given nucleotide ${r_i}$ at the position $j$ of the PFM $w$ and $P\left( {b,{r_i}} \right)$ is the background probability to observe the same nucleotide ${r_i}$

To characterize TBA scores across all our CREs, 5424 unique TF DNA-binding sites motifs from Jaspar (21), hPDI (22), SwissRegulon (23) and HOCOMOCO (24) public databases and from TRANSFAC Professional database (25) were collected in the form of PFM and mined.

TBA scores were computed for all TF PFMs across all tissue-specific and global CREs. For each combination of TF PFM and CRE, we computed both a TBA score considering the CRE sequence described by the human reference genome (hg19) and a set of TBA scores computed on all common alleles identified from 1000 Genomes Project individuals. Specifically, phased 1000 Genomes Project SNP genotypes (release 20130502; 2504 individuals) were first downloaded and combined with the human reference genome to build the overall landscape of alleles observed at the CRE. Then, allele frequencies were computed and only common alleles with an observed frequency >1% were retained and used to compute the fraction of common alleles with significant TF TBA score.

Statistical significance of a TBA score for a TF PFM at a specific CRE was calculated using a permutation approach. Considering the huge amount of TBA scores we computed across all global and tissue CREs (*n* = 5.6e10), an *ad hoc* strategy was needed to reduce the overall computational cost of TBA significance calculation. After determining the linear correlation that exists between TBA scores and target sequence lengths (Supplementary Figure 1), all computed TBA scores were normalized with respect to the corresponding CRE length and significance of the score was determined by comparing the TBA value against a PFM-specific reference distribution of normalized TBA scores computed across 100K random genomic regions of different lengths; 5424 reference distributions were computed, one for each TF PFM. In particular, TBA normalized score thresholds for different *P*-value cutoffs (from 5e-02 to 1e-05) were pre-computed from the reference distribution and rapidly used to determine TBA significance at the different cutoffs. Note that the *P*-value cutoff at 1e-05, which is set as default cutoff in CONREL, allows for stringent multiple hypothesis correction at a specific CRE; more relaxed *P*-value cutoffs can be set for exploratory analyses.

## Results

### Landscape of transcriptional CREs

Using more than 1000 ChIP-seq experiment data from ENCODE, CONREL provides an extensive collection of global and specific CREs for 38 tissue types and 198 different cell lines. CRE collections have been created considering narrow and broad peak data separately.

**Figure 2.**
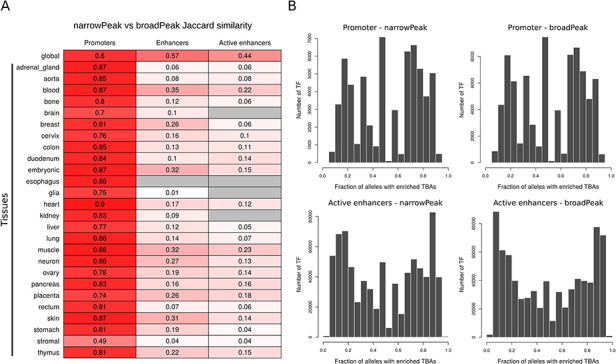
(A) Jaccard similarity of CREs derived from narrow versus broad peak data. Similarity is shown for global and tissue CRE promoters, enhancers and active enhancers. Intensity of red color is proportional to the similarity index, while gray color indicates no comparison available. (B) Distribution of the number of TF presenting a specific enrichment fraction across 1000 Genomes Project common alleles for CRE promoters and active enhancers. Since the majority of TFs present a TBA enrichment fraction equal to 1, only TFs with TBA enrichment fraction <1 are depicted in the distribution.

A summary of the number of global CREs and the percentage of genome spanned by the identified regions for promoters, enhancers and active enhancers for both narrow and broad peaks is reported in Table 1. The same statistics for tissue and cell line CREs are reported in Supplementary Table 2. Global CRE promoters span about 1% of the human genome with tissue- and cell-line-level CRE promoters that variably span from 0.27% to 0.67% of the genome. For CRE enhancers and active enhancers, instead, while global consensuses span, respectively, around 30%–40% and 15%–20% of the human genome, tissue- and cell-line-level CREs have a large variability, with consensuses spanning from 0.005% to 15% of the genome. Direct comparison between CREs obtained from narrow and broad peak data (Figure 2A) shows that while global CREs are reasonably similar across the different regulatory element types, tissue CREs have good similarities for promoters but poor similarities for enhancers and active enhancers. This directly reflects the low and variable number of experiments available for specific tissue types across narrow and broad data collections as single experiments are indeed available for some tissues.

**Table 1.** Number of consensus regulatory elements and corresponding fraction of the human genome covered for CONREL global consensus regions computed using both narrow and broad peak data and for ENCODE and RoadMap data collections

	Promoters		Enhancers		Active enhancers	
	No. of regions	%	No. of regions	%	No. of regions	%
Global narrowPeak	25 512	0.80	716 249	30.63	290 424	15.92
Global broadPeak	28 307	0.96	303 125	42.10	115 720	22.62
ENCODE	70 292	NA	399 124	NA	NA	NA
RoadMap	81 232	1.44	NA	NA	2 328 936	12.64

The length distributions of global CREs are reported in Supplementary Figure 2. Supplementary Table 3, instead, reports summary statistics of tissue- and cell-line-level CREs length distributions.

Table 1 also reports global regulatory element annotations from ENCODE and RoadMap projects. Of note, although we observe a variability in the number of globally annotated regions across the three annotations, the percentage of the genome covered by the regions is comparable. Importantly, CONREL is the only among the three annotations that provides data at tissue and cell-line levels.

### Global and allele-specific distribution of transcription binding affinities across CREs

A summary of the mean number of TF with enriched TBA per CRE and the percentage of regions with enriched TF for each type of CRE across different *P*-value cutoffs is reported in Table 2. The same statistics for tissue CREs are reported in Supplementary Table 4 and Supplementary Table 5, respectively.

**Table 2.** Mean number and range of TFs with enriched TBAs at promoter, enhancer and active enhancer CREs at different significance cutoff, and percentage of CREs with enriched TF TBAs (N = narrowPeak and B = broadPeak)

	TBA significance	Promoters		Enhancers		Active enhancers	
	*P*-value cutoff	Mean number of	CREs %	Mean number of	CREs %	Mean number of	CREs %
		TF (min, max)		TF (min, max)		TF (min, max)	
N	1e-02	281 (0, 694)	100	256 (18, 1098)	100	286 (23, 1066)	100
	1e-03	125 (0, 417)	99.9	94 (0, 806)	99.8	116 (0, 778)	99.8
	1e-04	76 (0, 310)	93.7	53 (0, 694)	84.2	70 (0, 663)	86.5
	1e-05	51 (0, 246)	83.9	34 (0, 618)	59	46 (0, 575)	68.5
B	1e-02	302 (30, 694)	100	431 (23, 1124)	100	522 (24, 834)	100
	1e-03	140 (0, 417)	100	231 (0, 837)	99.9	299 (0, 834)	100
	1e-04	86 (0, 310)	97.8	164 (0, 707)	93.3	218 (0, 713)	98
	1e-05	56 (0, 246)	94.7	123 (0, 630)	84.4	166 (0, 630)	95.6

Considering broad peak data, when using the most stringent statistical cutoff, we found enriched TBAs in about 95% of promoters, 85% of enhancers and 95% of active enhancers. Considering narrow peak data, instead, we observed lower percentages with enriched TBAs in about 80% of promoters, 60% of enhancers and 70% of active enhancers. Considering the more relaxed statistic, enriched TBAs were found in all CREs.

Across all common CREs alleles that we characterized using 1000 Genomes Project genotype data, we were able to identify TF TBAs that are enriched or depleted in only a fraction of alleles, highlighting the potential presence of allele-specific regulatory mechanisms. For example, considering global CREs, we found about 1% and 4% of regions, respectively, in promoters and active enhancers, with TF TBAs enriched in less than 10% of 1000 Genomes Projects common alleles using the most stringent significance cutoff. Complete distribution of TF TBAs enrichment in CRE promoters and active enhancers is shown in Figure 2B.

### Web-interface implementation and usage example

CONREL has been implemented in R v3.6.1 and R package ‘Shiny v1.3.2’ running on the ‘Shiny-server v1.5.12.933’ (http://www.rstudio.com/shiny). The interface is accessed through a web browser. Several R packages are utilized in the background processes, including ‘shinyDashBoardPlus’ for designing the interface, ‘TnT’ for generating the genome browser, ‘biovizBase’ and ‘GenomicFeatures’ for providing a set of utilities for genomic data, ‘EnsDb.Hsapiens.v75’ and ‘EnsDb.Hsapiens.v86’ for providing, respectively, genomic annotations for GRCh37 and GRCh38 human reference genome builds.

CONREL is deployed on a virtual server with 4GB RAM, 40 GB of disk and 2 CPUs running the Ubuntu 16.04 LTS Linux operating system and containerized in a Singularity image. The Singularity image is also available for the download, together with a set of configuration scripts, to run application on a local server. The web-interface source code is available at https://github.com/cibiobcg/CONREL.

**Figure 3.**
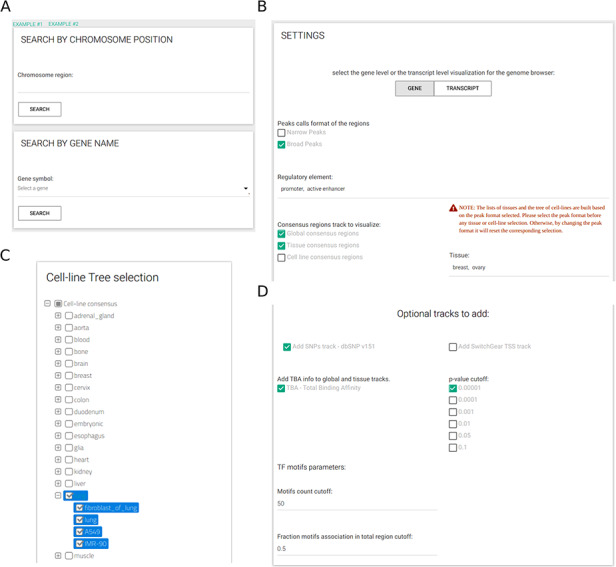
CONREL interface. (A) Search tab allows to select a specific genomic region or a gene name. Input tabs allow for the selection of source peak data and types of CRE to be displayed (B) using, when needed, also a cell line selection tree when activated (C) and TBA statistical filters (D).

The resource provides a user web-interface that, ones defined the gene name or the genomic region of interest (Figure 3A), requires to select at least three inputs (Figure 3B): (i) either narrow or broad peaks format, (ii) at least one type of regulatory element and (iii) at least one CRE (out of all available CREs). When cell-line CREs option is activated, a selection tree with specific cell lines divided by tissue of origin is shown and can be used to simplify the selection of all cell lines of interest (Figure 3C). All other inputs are optional. Easily selectable parameters include the TBA significance (default: 1e-05) and two parameters to select which TF PFMs to include in the analysis (Figure 3D); specifically, by setting the minimum number of sequences defining a PFM (default: 50) and the maximum fraction of CREs for an enriched PFM (default: 0.50), the user can avoid the inclusion of low-confidence PFMs that might be recurrently enriched across CREs.

**Figure 4.**
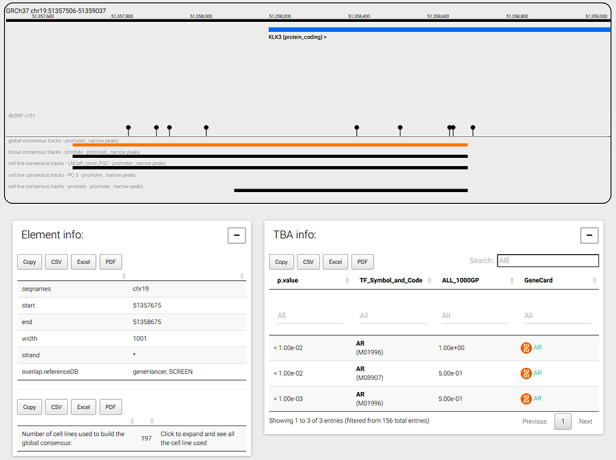
CONREL navigation web-page. The genome browser allows to navigate the selected genomic region or gene, while bottom panels allow to explore CRE information and TBA data. TBA data is shown in a multipage area where for each enriched TBA it is shown the significance at which the TF resulted enriched, and (when available) the fraction of 1000 Genomes Project common alleles that support the enrichment.

The genome browser tab is shown when a user selects a genomic coordinate or a gene symbol. As an example, Figure 4 displays the promoter region of the prostate-specific antigen, or KLK3 gene, the global CRE promoters and the tissue and cell line prostate CRE promoters. The genomic window can be navigated moving and zooming ±1 Mbp before and after the genomic coordinates loaded. Genes, transcripts and consensus regions can be selected to visualize more information. In particular, when a CRE is selected, the bottom panels show the genomic coordinates, the strand of the region, the number of experiments used to build the consensus and all information regarding TF TBA enrichments. In the example shown in Figure 4, TBA enrichments for androgen receptor (AR) PFMs are searched and found at significance cutoff less than or equal to 0.01 using the special ‘search’ functionality; of note, TBA enrichment of AR at that significance is mostly observed in only half of the common 1000 Genomes Project alleles, suggesting the importance of SNPs in shaping the specific PFM score across the promoter region. In addition, AR is a TF regulating KLK3 transcript levels and, interestingly, AR-negative prostate cancer PC3 cell line does not present a promoter annotation for KLK3 gene, hence indicating the absence of ChIP-seq signal supporting the presence of a promoter for KLK3 gene.

The interface further provides links to generate the DNA sequence of the displayed genomic window and to copy of download the selected consensus region information or the TBA info using different file formats (CSV, Excel or PDF).

### Comparison with other regulatory elements resources

In the absence of a ‘gold-standard’ to test our CREs, we decided to compare our global annotations against regulatory elements provided by other available resources. Specifically, we considered SCREEN (5), Ensembl (7) and GeneHancer (10) for promoters and EnhancerAtlas (8), DENdb (9), SCREEN, Ensembl and GeneHancer for enhancers. All regulatory region collections were converted to BED format and when needed coordinates were converted to human genome GRCh37 using liftOver tool and chain file from UCSC Genome Browser. We performed an asymmetrical pairwise comparison calculating for each resource pair two different coefficients: the percentage of regions of one resource that overlap the regions of the other resource and the ratio between the size of the genome covered by both resources divided by size of the genome covered by one or the other resource.

**Figure 5.**
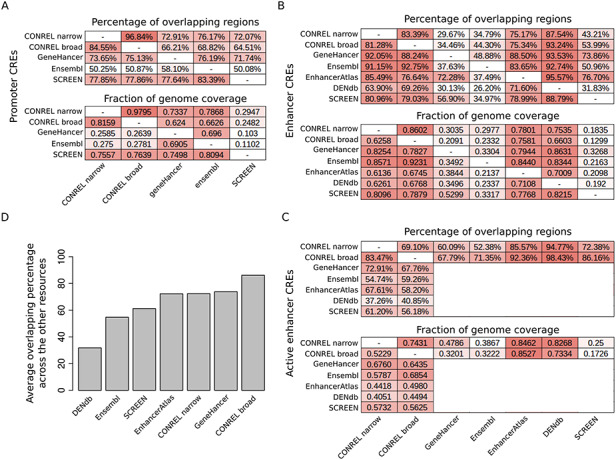
Comparison of CONREL CREs with other regulatory elements resources. (A) One-to-one comparison between CONREL, GeneHancer, Ensembl and SCREEN promoters. Each cell in the top matrix indicates the percentage of promoters annotated in the corresponding row resource that have an overlapping with the promoters annotated in the corresponding column resource. Each cell in the bottom matrix indicates the fraction of the genome covered by the promoters annotated in the corresponding row resource that is also captured by the promoters annotated in the corresponding column resource. (B) One-to-one comparison between CONREL, GeneHancer, Ensembl, EnhancerAtlas, DENdb and SCREEN enhancers. (C) One-to-one comparison between CONREL active enhancer and GeneHancer, Ensembl, EnhancerAtlas, DENdb and SCREEN enhancers. (D) Average percentage of CONREL active enhancer and GeneHancer, Ensembl, EnhancerAtlas, DENdb and SCREEN enhancers that have overlapping with each of the other resources.

As shown in Figure 5A, the pairwise comparison reveals an average of ∼75% promoters overlapping across all resource comparisons (excluding Ensembl), indicating an overall good concordance among the promoter annotations provided by most resources. In addition, genome coverage analysis reveals a level of concordance that reflects the differences in the size of the genome that is covered by the four annotations (∼1% for CONREL, ∼2% for GeneHancer and Ensembl and ∼0.3% for SCREEN, Supplementary Table 6). Of note, CONREL is the only resource providing promoter annotations at three resolution levels (global, tissue and cell line) and distinguishing between narrow and broad peak data-derived annotations. Indeed, while Ensembl provides global and also single experiments resolution levels, only global annotations are provided by SCREEN and GeneHancer.

Comparison of enhancer annotations reveals a good concordance between CONREL and the other resources, both in terms of overlapping and of common fraction of genome coverage (Figure 5B,C), but overall higher heterogeneity is observed among the different resources. While genome coverage of CONREL enhancers (∼30%) and active enhancers (∼20%) is more conservative with respect to EnhancerAtlas (∼55%) and DENdb (∼45%) enhancers (Supplementary Table 6), enhancers from SCREEN, GeneHancer and Ensembl are the most conservative ones, with an overall genome coverage of about 10%. While strong conservative annotations might reduce the presence of artifacts, the concordance between SCREEN, GeneHancer and Ensembl is not optimal, suggesting a potential divergence in the functional characterization of genomic regions/positions when these three resources are used. Of note, as shown in Figure 5D, CONREL active enhancers have on average the highest representation across all other resources. In addition, also in this case CONREL is the only resource providing enhancer annotations at three resolution levels and distinguishing between narrow and broad peak data-derived annotations.

To keep track of the relationships between our CREs and regulatory elements provided by the other resources considered here, we annotated our consensus regions with all the identified overlapping. Specifically, browsing global CREs with our web application will highlight which other resources provide support for the specific regulatory elements.

### Concordance of TBA annotations and transcriptional regulatory networks resources

**Figure 6.**
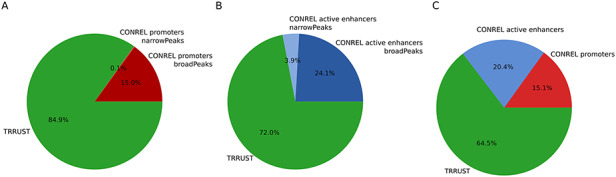
TRRUST transcriptional regulatory relationships captured by CONREL. (A) Cumulative fraction of TRRUST relationships captured by CONREL promoters, considering CREs from broadPeaks data and additional CREs from narrowPeaks data. (B) Cumulative fraction of TRRUST relationships captured by CONREL active enhancers, considering CREs from broadPeaks and additional CREs from narrowPeaks data. (C) Cumulative fraction of TRRUST relationships captured by CONREL promoters and additionally by active enhancers.

To investigate to what extent our TBA annotations are able to capture transcriptional regulatory networks, a list of manually curated transcriptional regulatory relationships from the sentence-based text mining TRRUST database (26) was retrieved, and only relationships involving TFs present in our data collection were retained. We then considered our global promoter and active enhancer CREs and extracted the list of closest protein-coding genes of all CREs enriching a motif of a TF present in TRRUST while considering the most stringent *P*-value cutoff (1e-05). Overall, TBA annotations of our global promoter CREs were able to explain about 15% of TRRUST relationships (Figure 6A). This percentage increased to 35.5% when also TBA annotations of our global active enhancer CREs were considered (Figure 6B,C). Although the number of relationships retrieved using CONREL TBA annotations is much higher (Table 2) with respect to the relationships described in TRRUST (∼7300), the percentage of TRRUST relationships that CONREL is able to capture is statistically significant. Specifically, by using a permutation approach where TRRUST regulators and targets are rewired randomly 1000 times, intersections observed in Figure 6 result statistically enriched both for CONREL global CREs promoters (*P* < 0.001 for both broad and narrow peak data) and CONREL global CREs active enhancers (*P* = 0.001 and *P* = 0.012, respectively, for narrow and broad peak data).

Overall, despite TRRUST and CONREL are built from very different input data, the obtained results suggest that CONREL can be also used to explore the topology of transcriptional regulatory networks.

## Conclusion

In this work, we presented CONREL, a web application that allows for the exploration of transcriptional CREs and that provides information about TF:DNA interactions through the use of TF TBAs. Built from ENCODE ChIP-seq peak data, CONREL provides an extensive collection of promoters, enhancers and active enhancers, defined by combining H3K4me1, H3K4me3 and H3K27ac histone markers. While many resources have been developed over the years to explore the landscape of regulatory elements from ENCODE ChIP-seq data, CONREL is the only resource aggregating ChIP-seq experiments at different abstraction levels, hence providing a unique collection of CREs for 198 cell lines and 38 tissue types, also combined to provide global consensuses. The low similarity we observed at tissue and cell-line levels for narrow versus broad peak CREs, on one hand, points out to the need of expanding the number of input experiments to better characterize consensus regions at these levels and, on the other hand, highlights the effectiveness of CONREL in integrating in a unique graphic environment CREs of different types, format and level of abstraction to more deeply explore the genomic structure of these regions.

For each regulatory element, the application provides collections of TFs that show enriched TBAs at different significance thresholds and can hence be used to elucidate regulatory mechanisms at specific regions. In addition, the landscape of TF TBA enrichment frequencies across common alleles in 1000 Genomes Project individuals is also provided for each regulatory element, allowing to identify TFs that might play a role in transcripts regulation only in a fraction of individuals. Further, the comparison with TRRUST database suggests that CONREL TBA annotations can be used to explore the structure and topology of transcriptional regulatory networks.

CONREL has been implemented as R Shiny application and provides a simple interface to navigate the data. Future development of the application will be devoted to the inclusion of additional ChIP-seq experiments from other resources to improve the confidence of CREs and the inclusion of additional transcriptional regulatory element types. In addition, we will also investigate approaches to improve the graphical interface experience to allow more insightful exploration of TF:DNA interactions.

## Supplementary Material

baaa071_Supp

## References

[1] Strahl B.D. and Allis C.D. (2000) The language of covalent histone modifications. *Nature*, 403, 41–45. 10638745 10.1038/47412

[2] Heintzman N.D., Hon G.C., Hawkins R.D. et al. (2009) Histone modifications at human enhancers reflect global cell-type-specific gene expression. *Nature*, 459, 108–112. 19295514 10.1038/nature07829 PMC2910248

[3] Creyghton M.P., Cheng A.W., Welstead G.G. et al. (2010) Histone H3K27ac separates active from poised enhancers and predicts developmental state. *Proc. Natl. Acad. Sci. U. S. A.*, 107, 21931–21936. 21106759 10.1073/pnas.1016071107 PMC3003124

[4] Rada-Iglesias A., Bajpai R., Swigut T. et al. (2011) A unique chromatin signature uncovers early developmental enhancers in humans. *Nature*, 470, 279–283. 21160473 10.1038/nature09692 PMC4445674

[5] ENCODE Project Consortium (2012) An integrated encyclopedia of DNA elements in the human genome. *Nature*, 489, 57–74. 22955616 10.1038/nature11247 PMC3439153

[6] Roadmap Epigenomics Consortium, Kundaje A., Meuleman W. et al. (2015) Integrative analysis of 111 reference human epigenomes. *Nature*, 518, 317–330. 25693563 10.1038/nature14248 PMC4530010

[7] Zerbino D.R., Wilder S.P., Johnson N. et al. (2015) The Ensembl Regulatory Build. *Genome Biol*, 16, 56. 10.1186/s13059-015-0621-5 PMC4407537 25887522

[8] Gao T., He B., Liu S. et al. (2016) EnhancerAtlas: a resource for enhancer annotation and analysis in 105 human cell/tissue types. *Bioinformatics*, 32, 3543–3551. 27515742 10.1093/bioinformatics/btw495 PMC5181530

[9] Ashoor H., Kleftogiannis D., Radovanovic A. et al. (2015) DENdb: database of integrated human enhancers. *Database*, 2015 bav085. 10.1093/database/bav085 PMC4560934 26342387

[10] Fishilevich S., Nudel R., Rappaport N. et al. (2017) GeneHancer: genome-wide integration of enhancers and target genes in GeneCards. *Database*, 2017, bax028. 10.1093/database/bax028 PMC5467550 28605766

[11] Griffon A., Barbier Q., Dalino J. et al. (2015) Integrative analysis of public ChIP-seq experiments reveals a complex multi-cell regulatory landscape. *Nucleic Acids Res.*, 43, e27–e27. 25477382 10.1093/nar/gku1280 PMC4344487

[12] Gheorghe M., Sandve G.K., Khan A. et al. (2019) A map of direct TF–DNA interactions in the human genome. *Nucleic Acids Res.*, 47, e21–e21. 30517703 10.1093/nar/gky1210 PMC6393237

[13] Tanay A. (2006) Extensive low-affinity transcriptional interactions in the yeast genome. *Genome Res.*, 16, 962–972. 16809671 10.1101/gr.5113606 PMC1524868

[14] Thomas-Chollier M., Hufton A., Heinig M. et al. (2011) Transcription factor binding predictions using TRAP for the analysis of ChIP-seq data and regulatory SNPs. *Nat. Protoc.*, 6, 1860–1869. 22051799 10.1038/nprot.2011.409

[15] Foat B.C., Morozov A.V. and Bussemaker H.J. (2006) Statistical mechanical modeling of genome-wide transcription factor occupancy data by MatrixREDUCE. *Bioinformatics*, 22, e141–e149. 16873464 10.1093/bioinformatics/btl223

[16] Ward L.D. and Bussemaker H.J. (2008) Predicting functional transcription factor binding through alignment-free and affinity-based analysis of orthologous promoter sequences. *Bioinformatics*, 24, i165–i171. 18586710 10.1093/bioinformatics/btn154 PMC2718632

[17] Quinlan A.R. and Hall I.M. (2010) BEDTools: a flexible suite of utilities for comparing genomic features. *Bioinformatics*, 26, 841–842. 20110278 10.1093/bioinformatics/btq033 PMC2832824

[18] Molineris I., Grassi E., Ala U. et al. (2011) Evolution of promoter affinity for transcription factors in the human lineage. *Mol. Biol. Evol.*, 28, 2173–2183. 21335606 10.1093/molbev/msr027

[19] Grassi E., Zapparoli E., Molineris I. et al. (2015) Total binding affinity profiles of regulatory regions predict transcription factor binding and gene expression in human cells. *PLoS One*, 10, e0143627. 10.1371/journal.pone.0143627 PMC4658012 26599758

[20] Grassi E., Mariella E., Forneris M. et al. (2017) A functional strategy to characterize expression Quantitative Trait Loci. *Hum. Genet.*, 136, 1477–1487. 29101457 10.1007/s00439-017-1849-9

[21] Khan A., Fornes O., Stigliani A. et al. (2018) JASPAR 2018: update of the open-access database of transcription factor binding profiles and its web framework. *Nucleic Acids Res.*, 46, D1284. 10.1093/nar/gkx1188 PMC5753202 29161433

[22] Xie Z., Hu S., Blackshaw S. et al. (2010) hPDI: a database of experimental human protein-DNA interactions. *Bioinformatics*, 26, 287–289. 19900953 10.1093/bioinformatics/btp631 PMC2804296

[23] Pachkov M., Balwierz P.J., Arnold P. et al. (2013) SwissRegulon, a database of genome-wide annotations of regulatory sites: recent updates. *Nucleic Acids Res.*, 41, D214–D220. 23180783 10.1093/nar/gks1145 PMC3531101

[24] Kulakovskiy I.V., Vorontsov I.E., Yevshin I.S. et al. (2018) HOCOMOCO: towards a complete collection of transcription factor binding models for human and mouse via large-scale ChIP-Seq analysis. *Nucleic Acids Res.*, 46, D252–D259. 29140464 10.1093/nar/gkx1106 PMC5753240

[25] Matys V., Kel-Margoulis O.V., Fricke E. et al. (2006) TRANSFAC and its module TRANSCompel: transcriptional gene regulation in eukaryotes. *Nucleic Acids Res.*, 34, D108–D110. 16381825 10.1093/nar/gkj143 PMC1347505

[26] Han H., Cho J.-W., Lee S. et al. (2018) TRRUST v2: an expanded reference database of human and mouse transcriptional regulatory interactions. *Nucleic Acids Res.*, 46, D380–D386. 29087512 10.1093/nar/gkx1013 PMC5753191

