# Supplementary material for: Exploring functionally annotated transcriptional consensus regulatory elements with CONREL
Source: Database (Oxford). 2020 Nov 9;2020:baaa071. doi: 10.1093/database/baaa071 (PMC7805434; doi:10.1093/database/baaa071)
Supplement: baaa071_Supp [file baaa071_supp.zip › SupplementaryFigures.docx]

**Supplementary Figures**

| **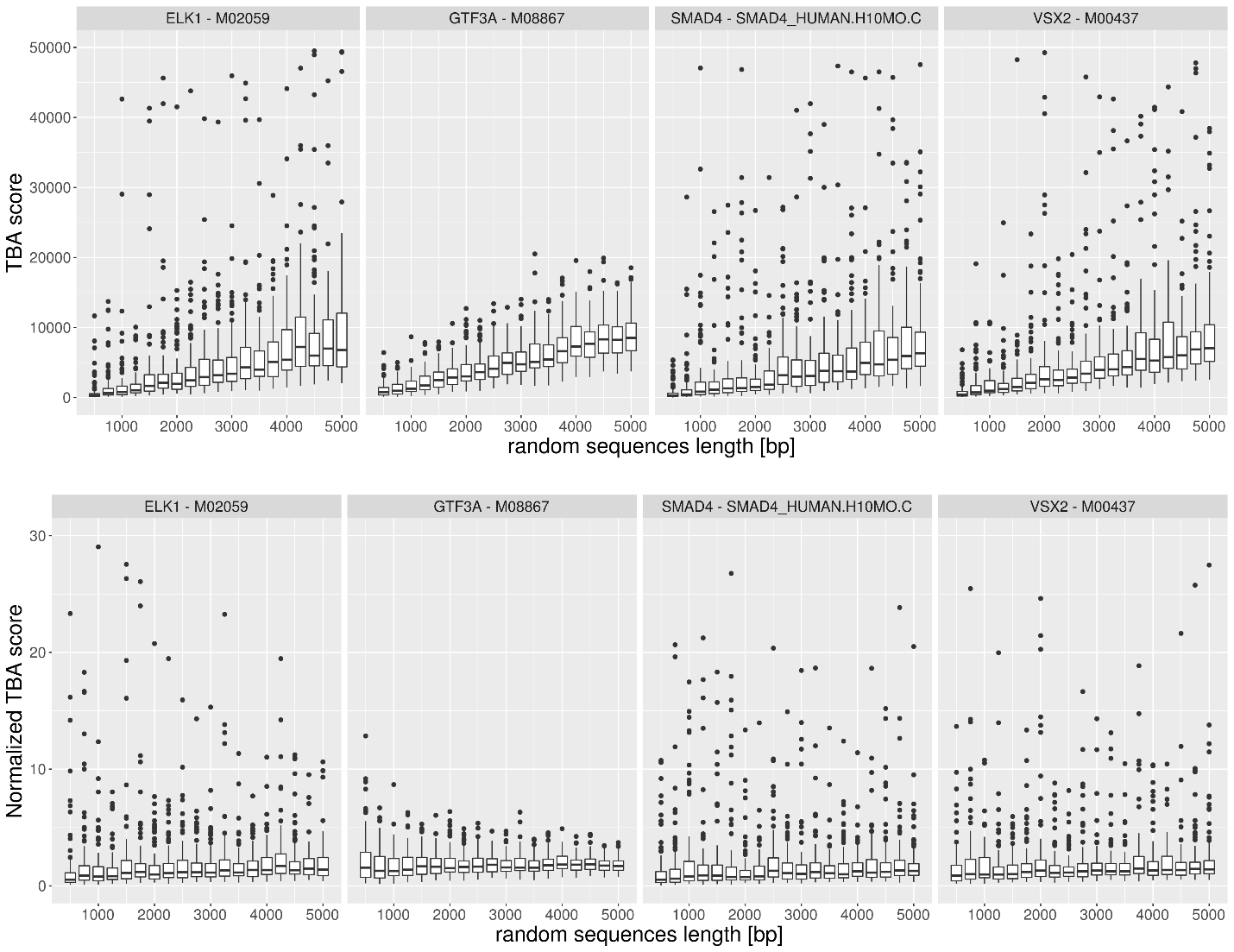** |
| --- |
| Supplementary Figure 1: Examples of TBA scores of different TF PFMs across random sequences of different lengths. Top row shows the linear relation that exists between sequence length and TBA scores. Bottom row shows TBA scores after sequence length normalization. |

| **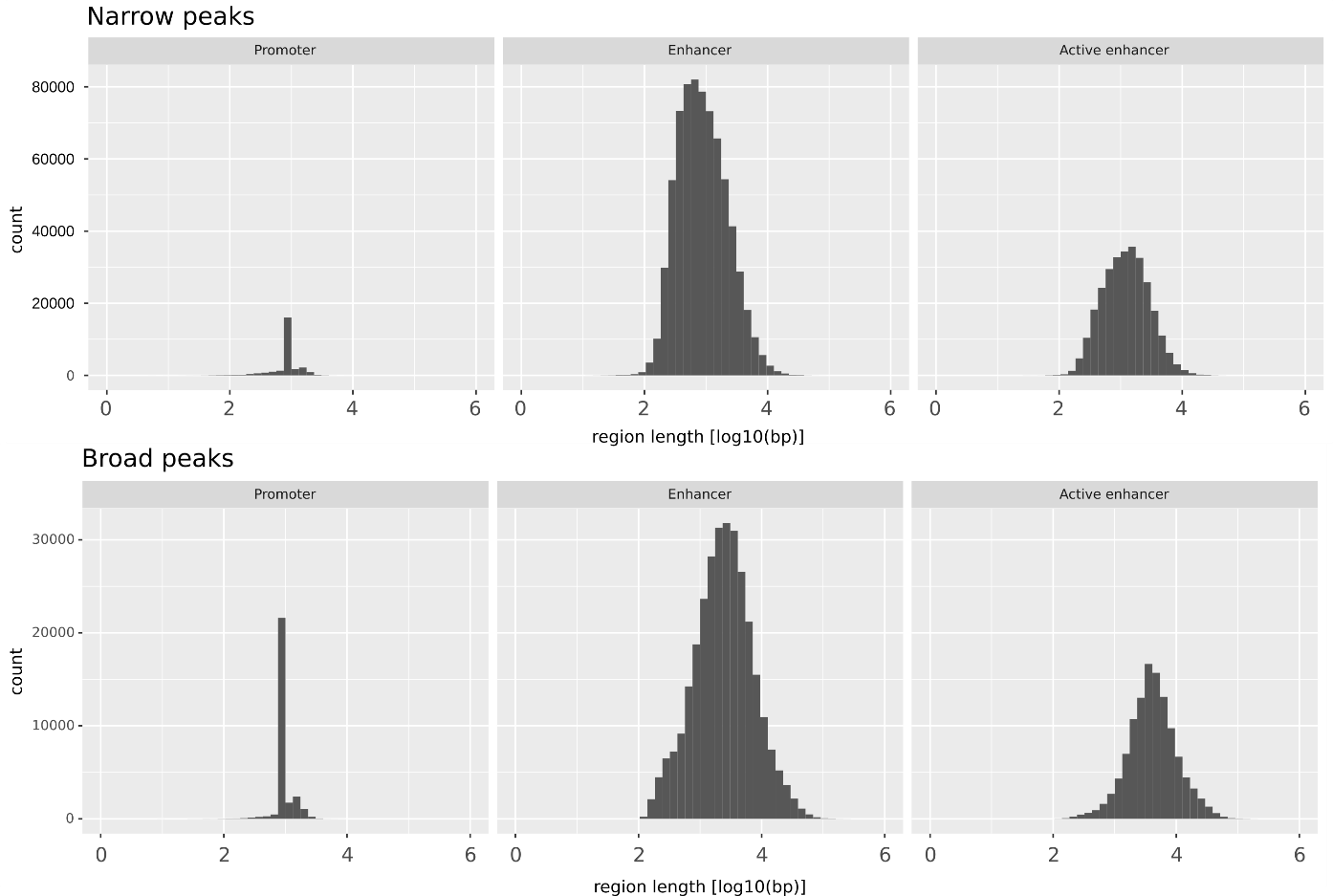** |
| --- |
| Supplementary Figure 2: Length distributions of global CRE promoters, enhancers and active enhancers using both narrow and broad peak data.. |
